# Intravascular ultrasound-factors associated with slow flow following rotational atherectomy in heavily calcified coronary artery

**DOI:** 10.1038/s41598-022-09585-z

**Published:** 2022-04-05

**Authors:** Hiroyuki Jinnouchi, Kenichi Sakakura, Yousuke Taniguchi, Takunori Tsukui, Yusuke Watanabe, Kei Yamamoto, Masaru Seguchi, Hiroshi Wada, Hideo Fujita

**Affiliations:** Division of Cardiovascular Medicine, Saitama Medical Center, Jichi Medical University, 1-847, Amanuma-cho, Omiya-ku, Saitama, Japan

**Keywords:** Interventional cardiology, Outcomes research, Cardiology, Medical research

## Abstract

Intravascular ultrasound (IVUS) can provide useful information in patients undergoing complex percutaneous coronary intervention with rotational atherectomy (RA). The association between IVUS findings and slow flow following rotational atherectomy (RA) has not been investigated, although slow flow has been shown to be an unfavorable sign with worse outcomes. The aim of this study was to determine the IVUS-factors associated with slow flow just after RA. We retrospectively enrolled 290 lesions (5316 IVUS-frames) with RA, which were divided into the slow flow group (n = 43 with 1029 IVUS-frames) and the non-slow flow group (n = 247 with 4287 IVUS-frames) based on the presence of slow flow. Multivariate regression analysis assessed the IVUS-factors associated with slow flow. Slow flow was significantly associated with long lesion length, the maximum number of reverberations [odds ratio (OR) 1.49; 95% confidence interval (CI) 1.07–2.07, p = 0.02] and nearly circumferential calcification at minimal lumen area (MLA) (≥ 300°) (OR, 2.21; 95% CI 1.13–4.32; p = 0.02). According to the maximum number of reverberations, the incidence of slow flow was 2.2% (n = 0), 11.9% (n = 1), 19.5% (n = 2), 22.5% (n = 3), and 44.4% (n = 4). In conclusion, IVUS findings such as longer lesion length, the maximum number of reverberations, and the greater arc of calcification at MLA may predict slow flow after RA. The operators need to pay more attention to the presence of reverberations to enhance the procedure safety.

## Introduction

Heavily calcified coronary lesions are still challenging for percutaneous coronary intervention (PCI) due to the high rate of target lesion failure, which is strongly associated with under-expansion^1,2^. In the calcified lesions, rotational atherectomy (RA) can provide benefits to achieve derivability of devices and further enlargement of the stent area^2,3^. During the RA procedure, there can be some specific complications, such as the slow flow phenomenon, vessel perforation, and burr entrapment^4,5^. Of these complications, slow flow just after RA is the most frequent complication^5^. Embolization of small particles originating from calcified lesions has been recognized as a major cause of slow flow after RA^4^. Although most slow flow is transient, even transient slow flow can lead to worse major cardiac adverse events^6,7^. Therefore, it is essential to understand clinical characteristics resulting in slow flow after RA. Previous studies have reported some predictors of slow flow such as lesion length and the burr-to-artery ratio^8,9^. Recently, our group showed unmodifiable (angulation and small reference diameter, etc.) and modifiable factors of slow flow (initial burr-to artery ratio and short single run, etc.)^5^.

To date, intra-coronary imaging devices such as intravascular ultrasound (IVUS) and optical coherence tomography (OCT) are widely used during PCI. Prospective multi-center trials have demonstrated that IVUS-guided PCI resulted in better clinical outcomes relative to angiography-guided PCI^10^. In addition, IVUS is helpful in predicting specific adverse events such as slow flow, stent edge dissection, and stent under-expansion during the procedures^11–13^. However, the association between IVUS findings and slow flow has not been investigated in calcified lesions requiring RA. The aim of this study was to determine IVUS findings which was associated with slow flow just after RA in heavily calcified coronary lesions.

## Methods

### Study population

This study was a single-center, retrospective observational study conducted at Saitama Medical Center, Jichi Medical University. Between January 2017 and December 2020, the consecutive patients undergoing PCI with RA were reviewed. The inclusion criteria were as follows: (1) RA was required, and (2) IVUS was performed. The exclusion criteria were as follows: (1) only OCT or optical frequent domain imaging was used instead of IVUS, (2) IVUS probe was manually pull-backed, (3) IVUS catheter was not passed even after RA, (4) IVUS image was difficult to analyze due to an air bubble in the catheter and (5) no IVUS frames with calcification of ≥ 180° and stenosis of ≥ 50%. The final study lesions were divided into the slow flow and non-slow flow groups according to the presence or absence of slow flow. Angiography immediately following RA was performed in every case, and the presence or absence of slow flow was recorded after every RA-procedure. Slow flow was defined as thrombolysis in myocardial infarction (TIMI) flow grade ≤ 2 without lesions leading to delayed filling of the distal vessels^14,15^. No-reflow was not separated from slow flow. This study was approved by the institutional review board of Saitama Medical Center, Jichi Medical University (S20-133). Written informed consent was waived because of the retrospective study design. All clinical information was obtained from a review of hospital records. All methods were performed in accordance with the relevant guidelines and regulations.

### Rotational atherectomy procedure

RA was performed using standard techniques as previously described^5^. Primary RA strategy was defined as RA without antecedent balloon dilatation. A nicorandil-based drug cocktail was infused into a targeted coronary artery through the RA catheter to avoid slow flow. IVUS was attempted after a 0.014-inch conventional guidewire was introduced into the target vessel and placed at a distal coronary segment. If initial IVUS catheter was not passed, a small RA burr or a small balloon was used. Following IVUS, the 0.014-inch conventional guidewire was replaced by a 0.009-inch guidewire (RotaWire floppy or RotaWire extra support guidewire; Boston Scientific, Marlborough, MA, USA) via a micro-catheter. Using the dynaglide mode, a RA burr was inserted to a position proximal to the lesion where initial rotational speed was set up within the conventional range (140,000–190,000 rpm). After activation, the spinning burr proceeded and turned back with a slow pecking motion. A single run was performed within 30 s, and the excessive speed down (> 5000 rpm) was avoided as much as possible. After crossing the lesion, the burr was removed using dynaglide mode or trapping balloon technique^16^. TIMI flow grade was evaluated by angiography immediately after the removal of the burr^9^. After IVUS images were acquired, the effect of RA on the lesion was assessed. A greater burr size of a burr was added if needed. Subsequently, the lesion was dilated using a non-compliant balloon, scoring balloon, or cutting balloon for stenting or drug-coated balloon.

### Definition of patient and lesion characteristics and complications

The patient and lesion characteristics were previously described^5^. Reference diameter and lesion length by angiography were analyzed using offline-software QAgio XA7.3 (MEDIS Imaging System, Leiden, The Netherlands). If baseline creatinine kinase (CK) levels were normal, periprocedural myocardial infarction (MI) was defined as an elevation of CK levels ≥ 2 times the upper limit of normal along with an elevation of CK-myocardial band (MB) levels above the upper limit of normal the day following PCI^17,18^. If the baseline CK levels were above the upper limit of normal, periprocedural MI was defined as an additional increase in CK levels the day following PCI^17,18^.

### IVUS image acquisition

We performed IVUS using either of the following systems: Opticross (Boston Scientific, Marlborough, MA, USA) and Altaview (Terumo, Tokyo, Japan). Opticross and Altaview systems were automatically acquired at a pullback rate of 1 mm/s (30 frames/s) and 3 or 9 mm/s (30 or 10 frames/s), respectively. All IVUS images were stored digitally and analyzed offline by RadiAnt DICOM Viewer Ver. 2020.2. (Medixant, Poznan, Poland).

### IVUS image analysis

Calcified lesions where RA was performed were analyzed. Angiography was carefully reviewed. Using the motion of the RA-burr and anatomical characteristics such as locations of branches and stenosis in the angiography, the range of IVUS where RA burr runs with the rotational speed of 140,000–190,000 rpm was estimated. The most proximal and distal IVUS frames with calcification of ≥ 180° and stenosis of ≥ 50% within the estimated range were identified. The range from the most proximal to distal IVUS frames was analyzed. The presence of the ablated area in IVUS after RA was not used to identify the range for IVUS analysis. Lesion length by IVUS was defined as the longitudinal length from the most proximal and distal frames with calcifications of ≥ 180° and stenosis of ≥ 50%. The target lesions were analyzed every 1 mm. The maximum arc of calcification was defined as the greatest arc of calcification in the lesion. Minimal lumen area (MLA) was defined as the smallest lumen area in the lesion. Mean diameter was defined as the average of the visually longest and shortest diameter in the lumen. The lumen area was measured by tracing the luminal surface.

**Figure 1:**
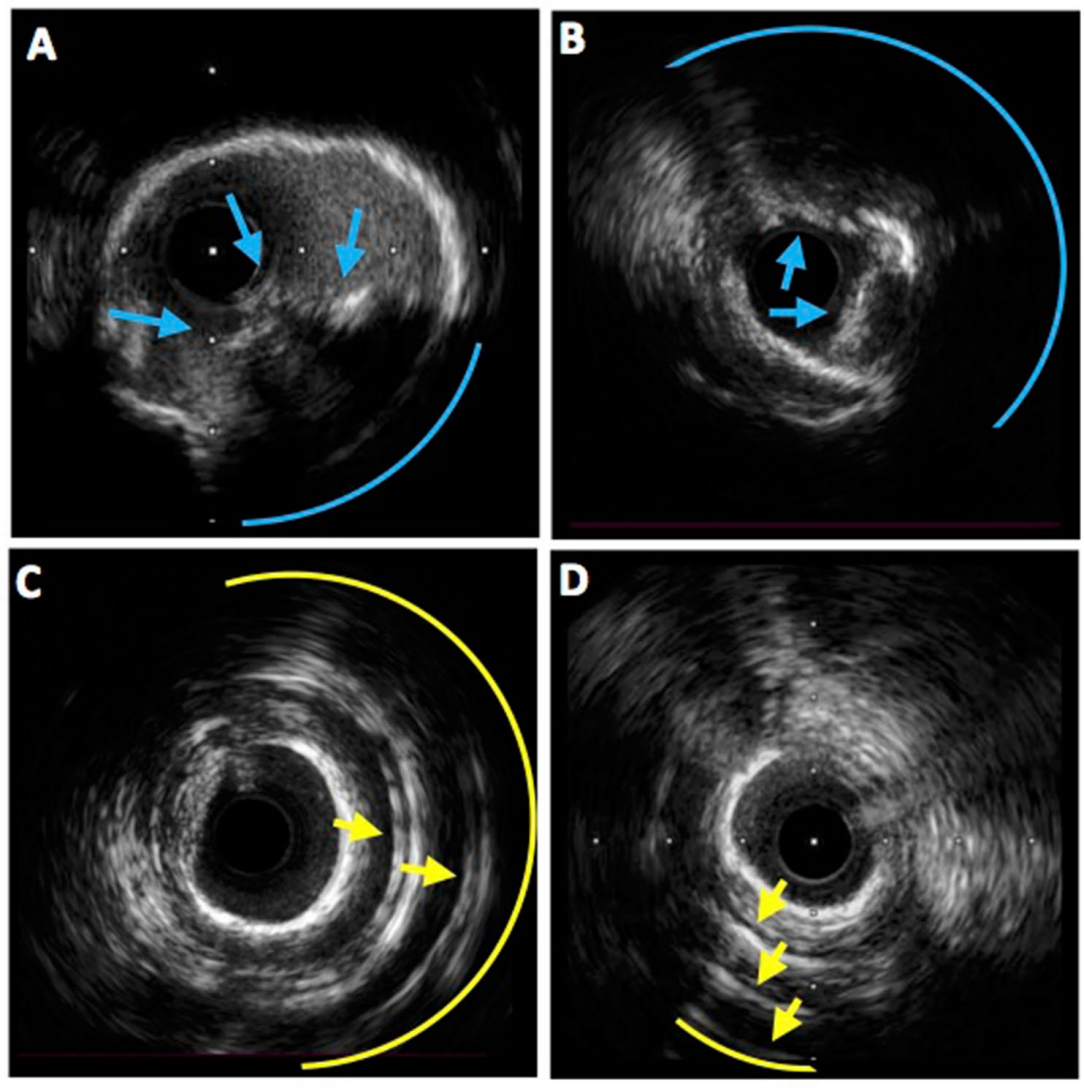
Evaluation of reverberation and calcified nodule. (**A**) Calcified nodule (blue arrows) showing irregular surface and convex shape with the distribution of one quadrant (blue arc). (**B**) A cross-sectional IVUS image showed calcified nodule (blue arrows) extending to two quadrants (blue arc). (**C**) A cross-sectional IVUS image showed two lines of reverberations (yellow arrows) with the distribution of nearly two quadrants (yellow arc). (**D**) There were three arctic lines of reverberations (yellow arrows) with the distribution of less than one quadrant (yellow arc). *IVUS* intravascular ultrasound.

When IVUS before RA was not available, IVUS after RA was used for analysis. When some IVUS images after different sizes of burr were available, the first IVUS image which could cross the entire lesion was used for the analysis to minimize an effect of the modification by RA. Either IVUS images before or after RA was used for the analysis. Therefore, IVUS images before and after RA were not compared. In such situations, a frame was considered to be affected by RA if the surface was artificially convex downward. In the frame, the affected lumen was divided into the ablated and the non-ablated areas. Initial luminal area was considered to be identical with the non-ablated area (Supplemental Fig. 1). Calcified nodule was defined as a convex-shaped calcification with an irregular surface^19,20^. The severity of calcified nodule was evaluated according to the following quadrant score: 0: 0°; score 1: > 0° and ≤ 90°; score 2: > 90° and ≤ 180°; score 3: > 180° and ≤ 270°; and score 4: > 270°; Fig. 1). Reverberation was defined as arc-shaped and high echoic lines behind calcifications which are typically equidistant (single or multiples) (Fig. 1)^21^. The number of reverberations was counted. The maximum number of reverberations was defined as the greatest number of reverberations in the analyzed lesion. The arc of reverberation was evaluated on the basis of the distribution of 0 to 4 quadrant scores as described above. At MLA, the arc of calcification, quadrant score of calcified nodule and reverberation, and the number of reverberations were also evaluated.

### Statistical analysis

Data are presented as values and percentages or median (interquartile range). Categorical variables were compared between the two groups with Fisher’s exact test. Based on the data distribution, continuous variables were compared between groups using the unpaired t-test or the Mann–Whitney U test. The data distribution was determined by the Wilk-Shapiro test. Of IVUS findings, the factors associated with slow flow just after RA were evaluated with multivariate stepwise logistic regression analysis. The multivariate logistic regression analysis with Wald Statistical criteria using the backward elimination method was performed. Variables with P < 0.05 in IVUS findings were used. Statistical analysis was performed using JMP version 10, and SPSS version 22. Two-sided P < 0.05 was considered to indicate statistical significance.

## Results

### Patient and lesion characteristics

**Figure 2:**
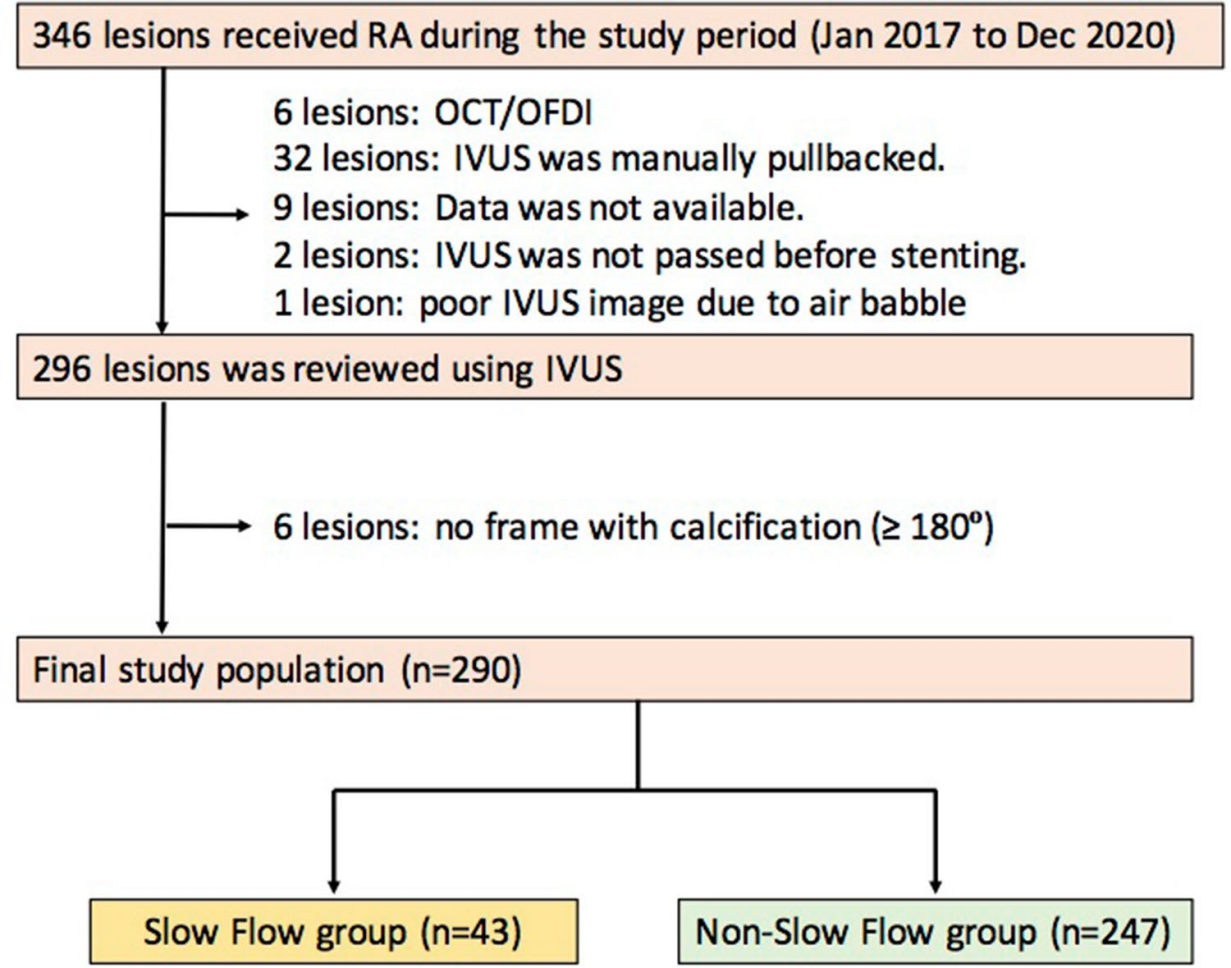
Study flow chart. *IVUS* intravascular ultrasound, *OCT* optical coherence tomography, *OFDI* optical frequent domain imaging, *RA* rotational atherectomy.

During this study period, 346 lesions underwent PCI with RA. Of these lesions, 290 which underwent IVUS before stenting were enrolled in this study (Fig. 2). Those lesions were divided into two groups: (1) the slow flow group (43 lesions) and (2) the non-slow flow group (247 lesions). The patient and lesion characteristics are listed in Table 1. The slow flow group showed a significantly smaller reference diameter, longer lesion, greater angulation of lesion by angiography, and lower pre-procedural TIMI flow grade relative to the non-slow flow group.

**Table 1:** Comparison of patients and lesions characteristics between the slow flow and non-slow flow groups.

	All (n = 290)	Slow flow (n = 43)	Non-Slow Flow (n = 247)	*p* value
**Patient characteristics**				
Age (years)	76 (70–81)	76 (70–82)	75 (70–81)	0.57
Men—n, (%)	210 (72.4)	32 (74.4)	178 (72.1)	0.85
Hypertension—n, (%)	282 (97.2)	43 (100)	239 (96.8)	0.61
Diabetes mellitus—n, (%)	167 (57.6)	27 (62.8)	140 (56.7)	0.51
Hyperlipidemia—n, (%)	271 (93.5)	42 (97.7)	229 (92.7)	0.33
Current smoker—n, (%) (n = 288)	50 (17.4)	9 (21.4)	41 (16.7)	0.51
Left ventricular ejection fraction (%)(n = 230)	59.6 (48.1–66.2)	59.4 (40.4–63.9)	59.6 (49.0–67.0)	0.17
Chronic renal failure on hemodialysis—n, (%)	76 (26.2)	8 (18.6)	68 (27.5)	0.26
Statin treatment—n, (%)	267 (92.1)	42 (97.7)	225 (91.1)	0.22
**Lesion characteristics**				
Culprit lesion in acute coronary syndrome—n, (%)	50 (17.2)	9 (20.9)	41 (16.6)	0.51
Culprit lesion in acute coronary syndrome with visible thrombus—n, (%)	1 (0.3)	1 (2.3)	0 (0)	0.15
Chronic total occlusion—n, (%)	2 (0.7)	1 (2.3)	1 (0.4)	0.28
In-stent lesion—n, (%)	14 (4.8)	1 (2.3)	13 (5.3)	0.70
**Target coronary artery**				0.83
Left main- left anterior descending artery—n, (%)	208 (71.7)	33 (76.7)	175 (70.9)	
Left circumflex artery—n, (%)	15 (5.2)	2 (4.7)	13 (5.3)	
Right coronary artery—n, (%)	67 (23.1)	8 (18.6)	59 (23.9)	
**Specific target coronary artery**				
Any ostial lesion—n, (%)	59 (20.3)	10 (23.3)	49 (19.8)	0.68
Reference diameter (mm)	2.4 (2.0–2.8)	2.0 (1.8 -2.4)	2.4 (2.1 -2.9)	< 0.0001
Lesion length (mm)	20.9 (11.7–34.7)	31.8 (17.8 -42.7)	20.3 (10.9 -33.8)	0.002
**Lesion angle**				0.002
Mild angulation (< 30°)	166 (57.2)	16 (37.2)	150 (60.7)	
Moderate angulation (30°–60°)	98 (33.8)	18 (41.9)	80 (32.4)	
Severe angulation (≥ 60°)	26 (9.0)	9 (20.9)	17 (6.9)	
Severe calcification, n (%)	287 (99.0)	43 (100)	244 (98.8)	1.00
**Pre-procedural TIMI-flow grade**				0.004
TIMI flow grade 3	269 (92.8)	35 (81.4)	234 (94.7)	
TIMI flow grade 2	16 (5.5)	5 (11.6)	11 (4.5)	
TIMI flow grade 1	4 (1.4)	2 (4.7)	2 (0.8)	
TIMI flow grade 0	1 (0.3)	1 (2.3)	0 (0)	

Values are presented as median (interquartile range) or n (%) for categorical variables.

*TIMI* thrombolysis in myocardial infarction.

### Procedural characteristics

The procedure details are summarized in Table 2. The primary RA strategy was significantly less used in the slow flow group compared to the non-slow flow group. Initial and final burr-to-artery ratios were significantly greater in the slow flow group than in the non-slow flow group. The total and mean single run times were significantly longer in the slow flow group than in the non-slow flow group.

**Table 2:** Comparison of procedural characteristics and outcomes between the slow flow and non-slow flow groups.

	All (n = 290)	Slow flow (n = 43)	Non-Slow Flow (n = 247)	*p* value
Primary RA strategy—n, (%)	276 (95.2)	36 (83.7)	240 (97.2)	0.002
Intra-aortic balloon pump support—n, (%)	5 (1.7)	1 (2.3)	4 (1.6)	0.56
Guidewire used during rotational atherectomy				< 0.0001
RotaWire floppy—n, (%)	221 (76.2)	22 (51.2)	199 (80.6)	
RotaWire extra support—n, (%)	46 (15.9)	11 (25.6)	35 (14.2)	
Guidewire switch from floppy to extra support—n, (%)	19 (6.6)	10 (23.3)	9 (3.6)	
Guidewire switch from extra support to floppy—n, (%)	4 (1.4)	0 (0)	4 (1.6)	
Number of burrs used	1 (1–1)	1 (1–2)	1 (1–1)	0.47
**Initial burr size**				0.61
1.25-mm	45 (15.5)	9 (20.9)	36 (14.6)	
1.5-mm	242 (83.5)	34 (79.1)	208 (84.2)	
1.75-mm	3 (1.0)	0 (0)	3 (1.2)	
**Final burr size**				0.51
1.25-mm	43 (14.8)	9 (20.9)	34 (13.8)	
1.5-mm	197 (67.9)	27 (62.8)	170 (68.8)	
1.75-mm	15 (5.2)	3 (7.0)	12 (4.9)	
2.0-mm	35 (12.1)	4 (9.3)	31 (12.6)	
Initial burr-to-artery ratio	0.63 ± 0.14	0.71 ± 0.12	0.61 ± 0.14	< 0.0001
Final burr-to-artery ratio	0.64 (0.55–0.73)	0.72 (0.61–0.86)	0.63 (0.54–0.71)	0.0001
Total run time (s)	61 (38–101)	109 (52–142)	57 (36–86)	< 0.0001
Mean single run time (s)	11.5 ± 2.6	12.7 ± 3.2	11.3 ± 2.4	0.0007
Mean rotational speed (× 1000 rpm)	177 (173–179)	178 (176–179)	177 (172–179)	0.14
Maximum speed reduction during RA (rpm) (n = 506)	6000 (4000–8000)	6000 (5000–10,000)	5000 (4000–8000)	0.02
Vasodilator drug, n (%)	84 (29.0)	30 (69.8)	54 (21.9)	< 0.0001
**Final procedure**				1.00
RA + balloon including drug-coating balloon—n, (%)	24 (8.3)	3 (7.0)	21 (8.5)	
RA + bare-metal stent—n, (%)	3 (1.0)	0 (0)	3 (1.2)	
RA + drug-eluting stent—n, (%)	263 (90.7)	40 (93.0)	223 (90.3)	

Values are presented as mean ± SD, median (interquartile range), or n (%) for categorical variables.

*IVUS* intravascular ultrasound, *RA* rotational atherectomy.

### Complications and in-hospital outcomes

Supplemental Table 1 summarizes the complications and in-hospital outcomes between the slow flow and non-slow flow groups. There were no significant differences in the final TIMI flow grade ≤ 2, periprocedural MI with slow flow, burr entrapment, and in-hospital death between the 2 groups. Of note, the overall incidence of final slow flow (TIMI flow grade ≤ 2) was very low (1.4%).

### IVUS assessment

**Figure 3:**
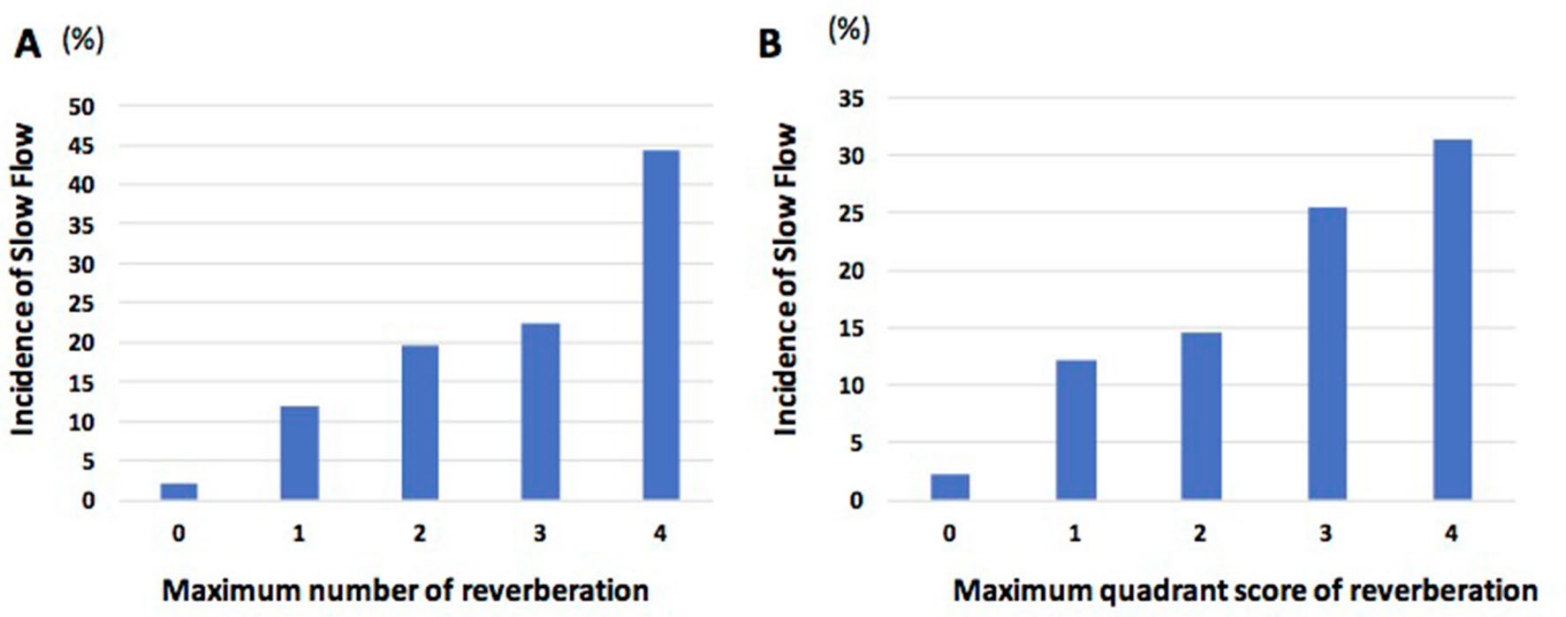
Association between reverberations and incidence of slow flow immediately after RA. (**A**) The graph showed the association between the maximum number of reverberations and slow flow. (**B**) The graph showed the association between the maximum quadrant score of reverberations and slow flow.

IVUS data are shown in Table 3. A total of 5316 frames from 290 lesions were analyzed (slow flow group, 1029 frames; non-slow flow group, 4287 frames). Lesion length measured by IVUS was significantly longer in the slow-flow group compared to the non-slow flow group. The maximum arc of calcification was significantly greater in the slow-flow group relative to the non-slow flow group. The slow flow group had a significantly smaller MLA relative to the non-slow flow group. Reverberation occurred more frequently in the slow-flow group than in the non-slow flow group. The maximum number and quadrant score of reverberation were significantly greater in the slow flow group than in the non-slow flow group. As the maximum number and quadrant score of reverberations increased, the incidence of slow flow increased (Fig. 3). At MLA, the slow flow group had a significantly greater arc of calcification, quadrant score of calcified nodule, number of reverberations, and quadrant score of reverberations compared to the non-slow flow group.

**Table 3:** Comparison of IVUS findings between the slow flow and non-slow flow groups.

	All (n = 290)	Slow flow (n = 43)	Non-Slow Flow (n = 247)	*p* value
Number of analyzed frames, n	5316	1029	4287	
IVUS before RA/ after RA, n (%)	164 (56.6)/126 (43.5)	14 (32.6)/29 (67.4)	150 (60.7)/97 (39.3)	0.0008
Mean affected area, mm^2^	0.44 (0.24–0.67) (n = 126)	0.45 (0.35–0.69) (n = 29)	0.42 (0.24–0.69) (n = 97)	0.47
Lesion length, mm	15 (8–26)	24 (12–36)	14 (7–24)	0.001
Maximum arc of calcification, °	360 (360–360)	360 (360–360)	360 (291–360)	0.02
Average of calcification-arc, °	244 (199–287)	246 (204–295)	242 (196–285)	0.33
Minimal lumen area, mm^2^	2.0 (1.6–2.6)	1.8 (1.5–2.3)	2.0 (1.6–2.7)	0.03
Mean diameter at minimal lumen, mm	1.6 (1.4–1.9)	1.5 (1.4–1.8)	1.6 (1.5–1.9)	0.06
Average of lumen area, mm^2^	3.5 (2.9–4.6)	3.4 (2.6–4.0)	3.5 (2.9–4.7)	0.12
Mean diameter, mm	2.1 (1.9–2.4)	2.1 (1.8–2.3)	2.1 (1.9–2.4)	0.14
Presence of calcified nodule, n (%)	123 (42.4)	16 (37.2)	107 (43.3)	0.51
Quadrant score of calcified nodule, n	0 (0–1)	0 (0–1)	0 (0–1)	0.45
Presence of reverberation, n (%)	246 (84.8)	42 (97.7)	204 (82.6)	0.01
Maximum quadrant of reverberation	2 (1–2)	2 (1–3)	1 (1–2)	0.003
Maximum number of reverberation	1 (1–2)	2 (1–2)	1 (1–2)	0.003
Arc of calcification at minimal lumen area, °	268 (206–360)	312 (226–360)	262 (204–333)	0.049
Quadrant score of calcified nodule at minimal lumen area, n	0 (0–0)	0 (0–0)	0 (0–0)	0.04
Quadrant score of reverberation at minimal lumen area, n	0 (0–1)	1 (0–1)	0 (0–1)	0.04
Number of reverberation at minimal lumen, n	0 (0–1)	1 (0–1)	0 (0–1)	0.04
The distance from IVUS to calcification at MLA, mm	0.59 (0.52–0.72)	0.57 (0.51–0.67)	0.60 (0.52–0.74)	0.11

Values are presented as median (interquartile range), or n (%) for categorical variables.

*IVUS* intravascular ultrasound.

### Factors associated with slow flow just after RA based on IVUS findings

Multivariate logistic regression analysis was used to evaluate the association between IVUS findings and slow flow (Table 4). Considering multi-collinearity, the presence of reverberation was not entered into the model since the presence of reverberation is similar to the number and quadrant score of reverberation. Two models were shown to avoid multicollinearity since variables at MLA were closely associated with the corresponding variables from overall frames. In the model 1, lesion length (≥ 24 mm) [odds ratio (OR), 2.43; 95% confidence interval (CI), 1.23–4.78; p = 0.01] and the maximum number of reverberations (every 1 increase) (OR 1.49; 95% CI 1.07–2.07; p = 0.02) were significantly associated with slow flow. In the model 2, lesion length (≥ 24 mm) (OR, 2.77; 95% CI 1.41–5.43; p = 0.003) and the arc of calcification at MLA (≥ 300°) (OR 2.21; 95% CI 1.13–4.32; p = 0.02) were significantly associated with slow flow.

**Table 4:** Multivariate logistic regression model to find factors associated with slow flow dependent variable: Slow flow (≤ TIMI-2) just after RA.

Independent variables	OR	95% CI	p value
**Model 1: Maximal arc of calcification, maximal number and quadrant score of reverberation were included as independent variables**			
Lesion length (≥ 24 mm)	2.43	1.23–4.78	0.01
Maximum number of reverberation (every 1 increase)	1.49	1.07–2.07	0.02
**Model 2: Arc of calcification, number and quadrant score of reverberation at MLA were included as independent variables**			
Lesion length (≥ 24 mm)	2.77	1.41–5.43	0.003
Quadrant score of calcified nodule at MLA (every 1 increase)	0.60	0.32–1.14	0.12
Arc of calcification at MLA (≥ 300°)	2.21	1.13–4.32	0.02

Multivariate stepwise logistic regression model to evaluate the association between IVUS findings and slow flow just after RA. Variables that had a significant association (p < 0.05) between the 2 groups were used as independent variables. Two models were shown since angle of calcification, the number and quadrant score of reverberation from all frames and MLA can lead to multicollinearity. The model 1 and 2 commonly included minimal lumen area (≥ 1.8 mm^2^), lesion length by IVUS (≥ 24 mm), and quadrant score of calcified nodule at MLA (every 1 increase). In the model 1, maximal angle of calcification (≥ 360°), maximal number and quadrant score of reverberation (every 1 increase). In the model 2, angle of calcification (≥ 300°), and number and quadrant score of reverberation at MLA (increase 1). The multivariate logistic regression analysis with Wald Statistical criteria using backward elimination methods was performed.

*CI* confidence interval, *IVUS* intravascular, *MLA* minimal lumen area, *OR* odds ratio, *RA* rotational atherectomy.

## Discussion

The main findings in the present study are summarized as follows; (1) the factors associated with slow flow were the maximum number of reverberations, longer lesion length, and a greater arc of calcification at MLA, (2) the number of reverberations correlated with the incidence of slow flow, (3) the slow flow group had significantly more complex IVUS-findings compared to the non-slow flow group (e.g., lesion length, MLA, the arc of calcification, the prevalence of reverberation and quadrant score of reverberations and quadrant score of calcified nodule at MLA). It is important to pay more attention to the factors associated with slow flow as described above so that we can stratify the potential risk of calcified lesions and plan an optimal strategy to prevent slow flow.

Previous intra-coronary imaging studies have investigated the prediction of slow flow in patients with acute coronary syndrome and stable angina not treated with RA^13,22^. The extent of lipid rich-plaques was associated with slow flow after stenting or ballooning^13,22^. Mechanism of slow flow is generally thought to result from microvascular embolization of atherosclerotic debris and associated thrombi, platelet activation, and release of vasoactive mediators^4,23^. However, the slow flow phenomenon can also be led by RA of calcified plaque^4,13^. To our best knowledge, this is the first study to investigate the association between intra-coronary imaging and slow flow after RA. The present study revealed that the maximum number of reverberations could be one of the predictors of slow flow just after RA. As the number of reverberations increased, the risk of slow flow after RA increased. Calcifications are typically described as high echoic signals with an acoustic shadow behind the signal by IVUS^21^. Additionally, reverberations that are concentric and arctic lines at equal intervals are frequently observed within the acoustic shadow^21,24^. The clinical importance of reverberations resulting from calcifications in coronary lesions has not been established.

Reverberations arises from the amplitude of ultrasound between echo-transducer and calcifications^24^. One portion of the reflected ultrasound usually returns to the transducer, whereas the other portion is reflected to calcification by the transducer^24^. Depending on the travel time of the wave, reverberations emerge as an artificial image within the acoustic shadow^24^. Therefore, the distance between reverberations is the same as the distance between the transducer and calcifications. If the wave is more completely reflected by calcifications, the initial wave traveling back to the transducer becomes more intense and the signals of reverberations are more highly emphasized. Component and thickness of calcifications affect the intensity of the reflected wave, and eventually the number and signal intensity of reverberations, since the thickness and purity of calcifications vary^25,26^. Considering the underlying mechanism, the severity of reverberations might be associated with the severity of calcifications such as thickness and components, which affect the number of particles by RA and influence the risk of slow flow.

Multivariate analysis revealed three factors associated with slow flow just after RA, i.e., the maximum number of reverberations, lesion length and the arc of calcification at MLA. In agreement with our IVUS study, previous studies also reported that lesion length measured by angiography was associated with the occurrence of slow flow after RA^8,9^. The arc of calcification at MLA was also a new factor associated with slow flow after RA. The greater arc of calcification at MLA would generate more vessel resistance, which leads to the difficulty of lesion-penetration by RA, although vessel resistance can be affected by many factors. The complex lesions with a greater arc of calcification at MLA generally result in longer total run time leading to an increase in particles and subsequently the risk of slow flow. Previous our study showed that the IVUS cross-ability was significantly associated with slow flow after RA^27^. The IVUS-uncrossable lesions had more complex lesion characteristics by angiography than the IVUS-crossable lesions, suggesting the association between the complexity of lesion and slow flow^27^. Using IVUS, the present study was successful in extracting the arc of calcification at MLA as an important factor for slow flow, since it is difficult to classify the severity of calcifications by angiography alone in a lesion requiring RA.

IVUS-guided PCI improved clinical outcomes and achieved greater stent-expansion than angiography-guided PCI^10^. IVUS can provide useful information to plan a strategy during the complex PCI. IVUS offer information such as the necessity of RA, adequate burr size, range of lesion to be ablated by RA and necessity of size-up of burr during PCI using RA^28^. In addition, the current study showed that IVUS findings may be useful for predicting the occurrence of slow flow just after RA. Thus, IVUS findings can facilitate risk stratification of slow flow just after RA. To prevent slow flow, the risk of slow flow can be shared among staff in the catheter laboratory team. More attention can be paid to vital signs or symptoms, and intra-coronary dilation drugs such as nicorandil or nitroprusside can be prepared. Furthermore, specific RA techniques such as halfway RA may be applicable to prevent slow flow in high-risk lesions^29^. As a result, these IVUS information may be helpful for the operators to plan safer PCI using RA.

### Study limitations

There were several limitations in this study. First, this study was a single-center, retrospective, and observational study. Second, the study population was small. Therefore, multi-center, prospective trials including the large population are warranted in order to confirm our results. Third, the incidence of slow flow was relatively high (14.8%), in part because we evaluated slow flow just after RA. Since both final slow flow and periprocedural MI with slow flow were rare (1.4% for final slow flow and 1.7% for periprocedural MI) in this study, we might pick up minor slow flow. Fourth, this study did not analyze the IVUS parameters associated with lipid components and the remodeling index which were recognized as the factors of slow flow in the patients with acute coronary syndrome^13,30^. However, the absence of lipid and the outline of vessels are not evaluable especially in the population requiring RA since images behind calcifications by IVUS are invisible. Fifth, the distance from IVUS to calcification was not a factor associated with slow flow. Although the volume of calcification ablated by RA might be associated with slow flow, not only the distance but also other factors such as a type of wire (soft or hard), lumen area and the size of burr complicatedly affect the volume of calcification ablated by RA. Sixth, determining the IVUS frames where RA ran was based on angiography showing the motion of RA burr. However, after determining the IVUS frames where RA ran, the most proximal and distal frames with calcifications of ≥ 180° and stenosis of ≥ 50% were identified to be analyzed. Therefore, the subjectiveness of the range to be analyzed was reduced. Seventh, we did not exclude the culprit lesions of acute coronary syndrome from the study population. The presence of a thrombus in acute coronary syndrome could be a cause of slow flow. However, emergent PCI using RA was avoided as much as possible, since emergent PCI using RA has been known as a risk of worse clinical outcomes^31^. Additionally, if RA was necessary in lesions with acute coronary syndrome, RA was usually performed after heparization for at least several days to reduce the burden of the thrombus. Therefore, the effect of a thrombus on slow flow was minimized. Eight, this study included lesions in which IVUS was not crossable before RA or ballooning as well as those in which IVUS was crossable before any procedure. In the lesions where the initial IVUS was not crossable, the IVUS findings were collected after RA or balloon, which suggests the presence of modification in the IVUS findings. In this study, reverberations were observed in many lesions before RA. However, some reverberations might be produced by RA. We could not discriminate reverberations produced by RA from natural reverberations. However, the lesions where IVUS was not crossable were more important than the lesions in which IVUS was crossable, since these lesions are the central issue of calcified plaques requiring RA. Therefore, it is essential to include the lesions in which IVUS was not crossable regardless of IVUS analysis before and after RA or balloon. Finally, the assessment of reverberations was performed regardless of the ablated area or non-ablated area. In the ablated area, the reverberations might result from ablation by RA. However, even if the ablated area was identified based on the presence of an artificially convex-downward surface, the possibility remained that an artificially convex-downward surface was present before RA.

## Conclusions

This IVUS study showed that the factors associated with slow flow just after RA were the maximum number of reverberations, lesion length and the arc of calcification at MLA. When using RA, it is helpful for the operators to pay more attention to these IVUS findings in order to recognize the risk of slow flow.

## Supplementary Information

Supplementary Figure 1.

Supplementary Information 1.

## Data Availability

All data are available from the corresponding author on reasonable request.

## Abbreviations

CI: Confidence interval
IVUS: Intravascular ultrasound
MLA: Minimal lumen area
OR: Odds ratio
PCI: Percutaneous coronary intervention
RA: Rotational atherectomy
