# Supplementary figures and images for: Intravascular ultrasound-factors associated with slow flow following rotational atherectomy in heavily calcified coronary artery
Source: Sci Rep. 2022 Apr 5;12:5674. doi: 10.1038/s41598-022-09585-z (PMC8983755; doi:10.1038/s41598-022-09585-z)

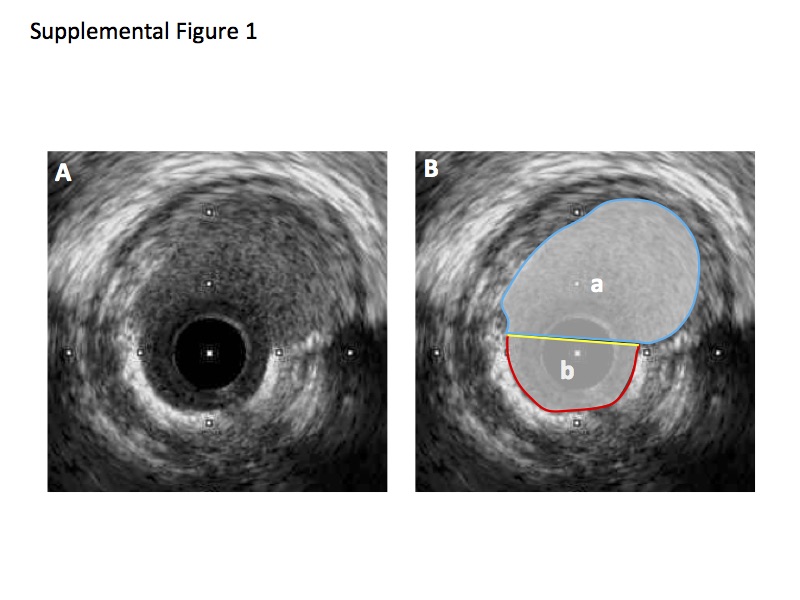

Supplement: Supplementary file 1 — Supplementary Figure 1. [file 41598_2022_9585_MOESM1_ESM.jpg]
