# Supplementary material for: Intravascular ultrasound-factors associated with slow flow following rotational atherectomy in heavily calcified coronary artery
Source: Sci Rep. 2022 Apr 5;12:5674. doi: 10.1038/s41598-022-09585-z (PMC8983755; doi:10.1038/s41598-022-09585-z)
Supplement: Supplementary file 2 — Supplementary Information 1. [file 41598_2022_9585_MOESM2_ESM.docx]

**Supplemental Table 1. Comparison of complications and outcomes between the slow flow and non-slow flow groups**

|  | All (n = 290) | Slow flow (n = 43) | Non-Slow Flow (n = 247) | *p* value |
| --- | --- | --- | --- | --- |
| Slow flow (≤ TIMI 1 just after RA– n, (%)) | 9 (3.10) | 9 (20.9) | 0 (0) | <0.0001 |
| Final TIMI flow grade ≤2 – n, (%) | 4 (1.4) | 2 (4.7) | 2 (0.8) | 0.11 |
| Periprocedural MI with slow flow – n, (%) | 5 (1.7) | 1 (2.3) | 4 (1.6) | 0.55 |
| Burr entrapment – n, (%) | 1 (0.3) | 0 (0) | 1 (0.4) | 1.00 |
| In-hospital death (irrespective of procedural complications) | 1 (0.3) | 1 (2.3) | 0 (0) | 0.15 |

Values are presented as n (%) for categorical variables. MI, myocardial infarction; TIMI, Thrombolysis in Myocardial Infarction.

**Supplemental Figure 1. Measurement of lumen area affected by rotational atherectomy. A.** Lower half part of the lumen showed superficial calcification ablated by rotational atherectomy. **B.** The IVUS image showed the non-ablated area (a, blue line) and the ablated area (b, red and yellow lines). Both edges of the ablated line (red line) were connected with a straight line (yellow line), since the original surface of the initial luminal area needs to be estimated. The ablated area was defined as an area enclosed by the ablated line (red line) and the straight line (yellow line).
